# A Rare Case of Statin-Induced Diplopia: An Often-Overlooked but Reported Side Effect

**DOI:** 10.7759/cureus.15117

**Published:** 2021-05-19

**Authors:** Randa Abdelmasih, Ramy Abdelmaseih, Justin Reed

**Affiliations:** 1 Internal Medicine, University of Central Florida College of Medicine, Ocala, USA

**Keywords:** atorvastatin, statin, hmg-coa reductase, diplopia, myopathy, side effect, adverse event

## Abstract

Statins are a class of medications indicated for atherosclerotic cardiovascular diseases and dyslipidemia. Ever since their introduction, various side effects have been reported with their use. Statin-induced myopathy is a well-established side effect of the medication, ranging in severity from mild myotoxicity to fatal rhabdomyolysis, with or without an increase in creatine kinase levels. Statin-induced diplopia, ptosis, or ophthalmoplegia are very rare, but they have been reported as adverse events in a handful of cases. These adverse events typically result from the progressive weakening of the external ocular musculature or the levator palpebrae superioris muscle. In this report, we present a rare case of statin-induced diplopia in a patient who had been on atorvastatin therapy for years. We believe this report will increase awareness among physicians about such an adverse event related to statins.

## Introduction

3-hydroxy-3-methylglutaryl-CoA reductase inhibitors (HMG-CoA reductase), commonly known as statins, constitute the first-line therapy for treating hypercholesterolemia. Their use has been progressively on the rise since 1992 in light of several studies on their role in primary and secondary prevention of coronary heart disease, stroke, and other causes of death associated with high cholesterol levels.

HMG-CoA reductase is the first and key-limiting enzyme in hepatic cholesterol synthesis. Statins work by competitively blocking this enzyme, thereby leading to a decreased conversion of HMG-CoA to mevalonic acid [1]. This in turn increases the production of microsomal HMG-CoA reductase and cell surface low-density lipoprotein (LDL) receptor expression. It is reported that statins can reduce up to 20-55% of circulating LDL-C levels [2]. Due to the inhibition of isoprenoid intermediates synthesis of the mevalonate pathway, statins have also shown some non-lipid-lowering effects, such as improving endothelial function, atherosclerotic plaque stabilization, as well as anti-inflammatory and immunomodulatory effects.

Statins are generally well-tolerated medications; however, many adverse events ranging from mild to severe in impact have been associated with their use. These adverse events include myalgia, myositis [3], rhabdomyolysis (0.1%), and elevated liver enzymes (1%). Moreover, some studies suggest that statins play a role in increasing the risk of diabetes mellitus, cancer, dementia, polyneuropathy, hemorrhagic stroke, and kidney disease. Statin-associated eye problems appear to be rare, but they have been reported as side effects. We discuss a case of statin-induced diplopia in a patient who had been on atorvastatin therapy for years.

## Case presentation

An 82-year-old male with a past medical history of coronary artery disease, hyperlipidemia on atorvastatin therapy for several years, type II diabetes mellitus, and hypertension presented to our facility with a complaint of double vision for two days. The condition was found to be continuous and progressive, with no alleviating or aggravating factors. The patient reported experiencing a similar episode four days prior, which had resolved without any intervention. The patient denied any history of trauma, eye pain, headache, loss of vision, slurred speech, recent infection, or muscle pain. On admission, his vital signs were stable. Physical exam showed visual acuity of approximately 20/30 without correction at distance with both eyes.

**Figure 1:**
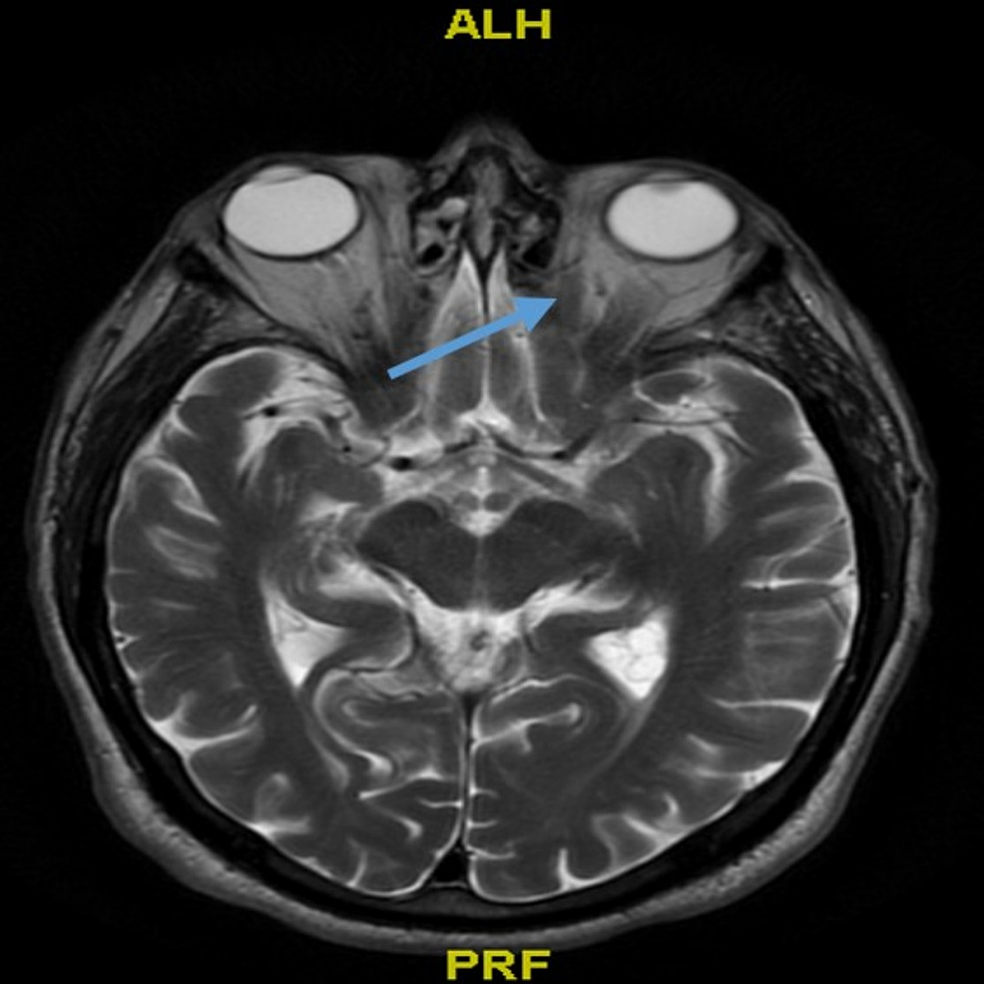
MRI brain with contrast The image shows extraocular myositis, which was more pronounced in the left medial rectus (blue arrow) MRI: magnetic resonance imaging

The patient's extraocular muscle movement appeared to be sluggish with vertical and horizontal diplopia. His cornea, anterior chamber, and pupils were normal in both eyes. His fundus examination showed no evidence of papilledema, hemorrhage, exudate, or retinal tear detachment. Laboratory workup revealed a normal thyroid-stimulating hormone (TSH) level of 1.580 mIU/L, erythrocyte sedimentation rate (ESR) of 8 mm/hr, and C-reactive protein (CRP) of <0.5 mg/dL. Anti-acetylcholine receptor antibody (AChR-Ab) was negative. CT brain was negative for any acute findings. A carotid duplex scan showed mild to moderate right extracranial carotid artery stenosis of 50-69% in the distal common carotid and the proximal external carotid artery, and left common extracranial carotid artery without any significant stenosis. MRI brain with contrast was concerning for extraocular myositis, which was more pronounced in the left medial rectus (Figure 1). Due to concern for statin-induced myopathy, coenzyme Q10 was checked and was found to be low at 0.53 ug/mL (normal range: 0.8-1.2 ug/mL). Atorvastatin was discontinued and oral coenzyme Q10 (CoQ10) 400 mg daily was started for two weeks with subsequent improvement in symptoms. The patient was discharged in a clinically stable condition with outpatient ophthalmology follow-up.

## Discussion

Diplopia refers to a symptom and is not a diagnosis on its own. Therefore, patients with diplopia should always undergo additional diagnostic studies to evaluate for underlying pathology and get treatment accordingly. There are two types of diplopia: (1) monocular diplopia in which the anterior segment of the eye is affected, such as cataracts, corneal problems, and astigmatism, and (2) binocular diplopia which indicates disconjugate alignment of the eyes. Binocular diplopia is usually a problem of the nerves or muscles as in cranial nerve palsy, brain stem lesions, orbital infiltration, thyroid diseases, myasthenia gravis, and as in our case, a medication side effect causing myositis [4]. Other important differential diagnoses for diplopia that should be considered and ruled out include stroke, cavernous sinus thrombosis causing ophthalmoplegia, orbital cellulitis, pseudotumor cerebri, intracranial mass or malignancy, subdural or subarachnoid hemorrhage, and orbital floor fractures.

Statin-induced diplopia is an extremely rare but occasionally reported side effect with a prevalence of 0.1%, while the concomitant use of gemfibrozil raises this figure to 0.5-2.5% [4]. Mizranita et al. have reported that 1.8% of all statin-associated events recorded by the Food and Drug Administration (FDA) between 1988 and 2011 were adverse ocular events. The authors further concluded that among all statins, atorvastatin was associated with the highest incidence of ocular side effects [5].

Statin-induced diplopia appears to be correlated to myositis of the extraocular muscles or mitochondrial dysfunction. The following etiologies may be associated with the development of statin-induced diplopia: reduction of ubiquinone or coenzyme Q10 through the inhibition of one of the key steps in coenzyme Q10 synthesis, leading to mitochondrial dysfunction with subsequent failure to replace damaged muscle proteins via the ubiquitin pathway. Other proposed mechanisms include reduced sarcoplasmic reticular cholesterol, calcium metabolism disruption in the skeletal muscles, and induction of muscle fibers apoptosis. In some cases, anti-acetylcholinesterase antibodies are elevated, which suggests an immune-mediated process. It is unclear if neuropathy contributes to diplopia [6].

Coenzyme Q10, or ubiquinone, is part of the mitochondrial electron transport system playing a crucial role in oxidative phosphorylation and adenosine triphosphate (ATP) production. Decreased coenzyme Q10 levels could potentially result in reduced rates of ATP production, resulting in myalgia or myopathy. Moreover, decreased coenzyme Q10 concentration may also lead to defective cell division and apoptosis, and enhanced free radical production leading to subsequent mitochondrial DNA damage. Several studies have demonstrated that statins decrease serum coenzyme Q10 levels, including atorvastatin, lovastatin, simvastatin, and pravastatin. Atorvastatin treatment was associated with a rapid drop in serum coenzyme Q10 levels after 14 days of therapy initiation, and this drop was even more pronounced after 30 days of therapy initiation. Banach et al. conducted a meta-analysis by pooling data from six randomized clinical trials including 450 patients to assess the significance of statins' effect on plasma coenzyme Q10 levels. The meta-analysis showed a significant reduction in plasma coenzyme Q10 levels following statin therapy initiation, regardless of their formulations, duration, or dose [7].

The drop in plasma coenzyme Q10 concentration after statin therapy initiation may be secondary to its lipid solubility and the reduction of LDL, which is a coenzyme Q10 man carrier in plasma. Other molecular-level mechanisms include the inhibition of coenzyme Q10 synthesis through interference with the production of mevalonic acid, which is a precursor of coenzyme Q10 synthesis. Excessive consumption of coenzyme Q10 may also play a role [6].

Currently, coenzyme is neither FDA-approved nor universally recommended for preventing statin-induced myopathy, as the data supporting its efficacy are equivocal, with some studies demonstrating favorable outcomes with coenzyme Q10 supplementation reducing the incidence and severity of statin-induced myopathy, while other studies showing no beneficial effects of supplementation [8]. A recently published randomized clinical trial by Skarlovnik et al. that included 50 patients with statin-induced myopathy showed that coenzyme Q10 supplementation - 50 mg twice/day - showed a significant reduction in rates of statin-induced mild to moderate muscular symptoms, resulting in increased ability to perform daily activities [9]. Other data suggest that supplementation of coenzyme Q10 at a dose of 100-600 mg once/daily has shown to reduce statin-induced myopathy without noted side effects [10]. Upon literature review, we have observed that the statin-induced myopathy recovery profile is not well established. Some studies suggest complete recovery of statin-induced muscle adverse events after cessation of therapy, while others report variable persistent symptoms in 68% of patients despite therapy cessation. However, no piece of evidence has indicated a timeline window between statin therapy cessation or coenzyme Q10 supplementation and the resolution of symptoms.

This case highlights the importance of the early consideration of statin-induced diplopia in patients with unexplained external ophthalmoplegia. Although the timing of the initiation of statin therapy in relation to the subsequent adverse event can be suggestive, there is no established time frame in which the adverse events are more or less likely to occur. In our case, it happened after years of therapy. The treatment approach toward statin-induced diplopia basically involves the discontinuation of statin. Although controversial, some patients might benefit from CoQ10 supplementation in case of evidence showing low CoQ10. Most of the symptoms are reversible upon the discontinuation of statin.

## Conclusions

Given the widespread use of HMG-CoA reductase inhibitors among various populations, we believe it is important for clinicians to be familiar with this rare but reported adverse event of statin use. Additionally, this report emphasizes the importance of meticulous and careful history taking, including a review of medications, when dealing with a patient with unexplained diplopia. Further studies are required to gain a better understanding of the role of coenzyme Q10 in statin-induced myopathy and the benefit of its supplementation in the prevention and mitigation of this adverse event.
